# Isolation, Purification, Structural Characterization of Acidic Polysaccharides from *Brassica rapa* L. Rhizomes and Their In Vitro Activity Verification in Ameliorating Glycolipid Metabolism Disorders

**DOI:** 10.3390/foods15071152

**Published:** 2026-03-27

**Authors:** Sanawar Mansur, Xuhan Fang, Ting Li, Aytursun Abuduwaili, Ahmidin Wali, Anargvl Mahmut, Kailibinuer Abulaiti, Zulfiye Talat, Weihao Wang

**Affiliations:** 1 College of Chemistry and Chemical Engineering, Xinjiang Agricultural University, Urumqi 830052, China; sanam0405@163.com (S.M.); fangxuhan@outlook.com (X.F.); 18234819874@163.com (T.L.); ayituxunabuduwaili14@mails.ucas.ac.cn (A.A.); 2 State Key Laboratory of Xinjiang Indigenous Medicinal Plants Resource Utilization, Xinjiang Technical Institute of Physics and Chemistry, Chinese Academy of Sciences, Urumqi 830011, China; ahmidin@ms.xjb.ac.cn; 3 Xinjiang Institute of Materia Medica, Urumqi 830011, China; 18999966874@163.com (A.M.); 13139669862@163.com (K.A.); 4 Institute of Chinese Materia Medica, China Academy of Chinese Medical Sciences, Beijing 100700, China

**Keywords:** *Brassica rapa* L., acidic polysaccharides, galacturonic acid, structure characterization, ameliorating glycolipid metabolism disorders

## Abstract

Acidic polysaccharides, valued for their outstanding bioactivity and physicochemical properties, represent a promising strategy for metabolic disease intervention. In this study, three acidic polysaccharide fractions (BRP-1, BRP-2, and BRP-3) were isolated from *Brassica rapa* L. using membrane filtration and ion-exchange chromatography. BRP-3, notable for its high galacturonic acid content (76.64%), was further purified to yield the homogeneous fraction BRP-3-1 (Mw = 22.3 kDa). Combining GC-MS, FTIR, and NMR analyses, we report for the first time the detailed structure of BRP-3-1—a heteropolysaccharide composed of rhamnose (1.687%), galacturonic acid (75.584%), galactose (14.452%), and arabinose (8.277%)—with a backbone composed with T-α-L-Ara*f*-(1 → 5)-α-L- Ara*f* -(1 → 4)-α-D-Gal*p*A-(1 → 4)-α-D-2-O- Gal*p*A Me-(1 → 4)-α-D-Gal*p*A-(1 → 4)-α-D-Gal*p*A-(1 → 3)-Galp-(1 → 4)-α-D-Gal*p*A, and T-Rha*p,* T-Gal*p* as well as T-Gal*p*A for branched chain and terminals. In HepG2 insulin-resistant cells, BRP-3-1 demonstrated potent dual regulation of glucose and lipid metabolism—enhancing glucose consumption, lowering total cholesterol, and significantly reducing triglyceride levels in the high-dose group (800 μg/mL), outperforming BRP-2. This work systematically defines the structure of a highly bioactive acidic polysaccharide from *B. rapa* L. and confirms its metabolic regulatory effects, offering a strong scientific foundation for its application in functional foods and as an adjuvant therapeutic for metabolic disorders.

## 1. Introduction

Turnip (*Brassica rapa* L.) is an economically important crop belonging to the genus Brassica within the Brassicaceae family and is widely cultivated across Europe, the Americas, and Asia. In traditional folk medicine, *B. rapa* L. has long been used to alleviate asthma, relieve cough, combat hypoxia, and reduce fatigue [1]. Modern pharmacological studies have further validated that *B. rapa* L. roots exhibit a broad spectrum of biological activities, including antibacterial, antioxidant, anticancer, anti-inflammatory, cardioprotective, hypolipidemic, hepatoprotective, antidiabetic, nephroprotective, and analgesic effects [2,3]. Phytochemical investigations have identified saponins, alkaloids, flavonoids, amino acids, and polysaccharides as the major bioactive constituents of *B. rapa* L. [2]. Among these, *B. rapa* L. polysaccharides—particularly abundant in the roots and stems—have attracted considerable attention due to their diverse and potent biological functions. Accumulating evidence demonstrates that these polysaccharides exert significant effects through a multi-target, network-based regulatory mechanism, contributing to immune activation [4,5], tumor suppression [6], metabolic regulation [7,8,9,10], and anti-fatigue activity [11]. Consequently, *B. rapa* L. polysaccharides have emerged as a key functional matrix for the innovative development of natural health products and therapeutics.

Acidic polysaccharides represent an important class of polysaccharide structural types and have become a frontier and hotspot in the field of glycobiology. The selection of extraction and purification methods profoundly influences the yield, structural characteristics, and biological activities of the obtained polysaccharides. Among various separation techniques, hot water extraction and ultrasonic-assisted extraction are currently the most commonly used methods for polysaccharide extraction. Zhao et al. [12] employed ultrasonic extraction combined with gel permeation chromatography to isolate four polysaccharide fractions from *B. rapa* L., designated as BRP-20, BRP-40, BRP-60, and BRP-80. Among these, BRP-40 and BRP-60 were acidic polysaccharides, while BRP-20 and BRP-80 were neutral polysaccharides. Monosaccharide composition analysis revealed that BRP-20 and BRP-40 primarily consisted of glucose, with no glucuronic acid detected. BRP-60 was mainly composed of galactose (37.33%), rhamnose (28.65%), and glucose (21.67%). In contrast, BRP-80 contained glucose (31.28%), galactose (25.71%), rhamnose (21.39%), and mannose (19.82%), with no xylose detected. Pharmacological activity evaluation demonstrated that all four fractions exhibited significant anti-fatigue activity. In another study, Cao et al. [10] obtained a neutral polysaccharide with a molecular weight of 31.378 kDa through ultrasonic-assisted extraction followed by ion-exchange column chromatography. This polysaccharide was composed of mannose and glucose in a molar ratio of 7.62:1 and displayed strong antioxidant and blood glucose-regulating activities in vitro. Structure-activity relationship studies indicated that a high uronic acid content and characteristic monosaccharide composition—particularly the presence of rhamnose—play a decisive role in conferring immunomodulatory and antioxidant activities to *B. rapa* L. polysaccharides [6,13,14,15]. These findings provide a theoretical basis for the targeted isolation and screening of polysaccharide fractions with high bioactivity.

This study employed an integrated strategy of “dialysis enrichment-ion exchange fractionation-gel filtration purification” to successfully isolate three acid polysaccharide fractions rich in galacturonic acid (BRP-1 to BRP-3) and a highly purified homogeneous fraction BRP-3-1 from *B. rapa* L. for the first time. Precise structural characterization of BRP-3-1 was achieved through multidimensional spectroscopic techniques, and its efficacy in regulating glycometabolic and lipid metabolism was validated in a human HepG-2 insulin resistance model. Results indicate that BRP-2 and BRP-3-1 synergistically enhance glucose consumption while reducing cholesterol and triglyceride accumulation, providing structurally defined, functionally characterized lead compounds for developing functional foods and drugs targeting dysregulated glycometabolism. This study not only enriches the structural and activity database of *B. rapa* L. acid polysaccharides but lays a solid experimental foundation for their in-depth development and subsequent research into their underlying mechanisms of action.

## 2. Materials and Methods

### 2.1. Material

*Brassica rapa* L. used in this study was harvested from Aqiale Town, Keping County, Xinjiang Uygur Autonomous Region, China. The species identification was performed by Associate Professor Zulfiye Talat, who is affiliated with Xinjiang Institute of Materia Medica, Urumqi, China, in September 2023. The voucher specimen is deposited in the Xinjiang Institute of Materia Medica, with specimen number: 652822-120602-092 (B) and herbarium code: XTNM.

### 2.2. Extraction, Separation and Purification of Polysaccharides

#### 2.2.1. Extraction of Polysaccharides

Fresh *Brassica rapa* L. were thoroughly cleaned by removing roots, leaves, and peel, then chopped and air-dried. The dried materials were ground into powder and sieved through a 20-mesh screen. The resulting powder was subjected to a single degreasing step with petroleum ether at a solid-to-liquid ratio of 1:5 (*w*/*v*, Kg/mL). Subsequently, two rounds of decolorization were performed using 95% ethanol. The defatted powder was naturally air-dried prior to further analysis.

Take 500 g of defatted and decolorized *B. rapa* L., add 10 L of deionized water, and reflux extract at 90 °C for 4 h. Filter under vacuum. Repeat the extraction twice under the same conditions and combine the two filtrates. Concentrate the mixture by heating, cool to room temperature. Add three-fold volume of anhydrous ethanol and let stand overnight. Centrifuge, dry the supernatant at 45 °C, and set aside for later use.

Resuspend 20.0 g of the dried solid in deionised water. Dialyse using a dialysis bag with a molecular weight cut-off of 8000–14000 Da, exchanging water every 24 h. Freeze-dry the dialysate to obtain *B. rapa* L. rhizome polysaccharides (BRPs).

#### 2.2.2. Isolation of Polysaccharides

Weigh 4.0 g of BRPs and dissolve in 10 mL of deionised water. After centrifugation at 8000 rpm for 10 min, the supernatant was collected and loaded onto a DEAE Sepharose Fast Flow anion-exchange resin column for separation. Elution was performed sequentially with deionized water, 0.2 M, 0.4 M, 0.6 M, and 0.8 M NaCl solutions at a flow rate of 2.0 mL/min, with 1.5 bed volumes (BV) eluted for each solution. Fractions were collected automatically (12 mL per tube) and monitored using the an-throne-sulfuric acid method. Elution curves were constructed with the number of effective tubes as the x-axis and absorbance at 620 nm as the y-axis. Fractions corresponding to the highest polysaccharide peaks were pooled, dialyzed against running water for 24 h using a dialysis bag with a 3500 Da molecular weight cutoff, and concentrated by heating. The resulting solution was freeze-dried to obtain different polysaccharide fractions (BRP-1, BRP-2, BRP-3).

#### 2.2.3. Purification of Polysaccharides

Weigh 50 mg of BPR-3 and dissolve in 5 mL of deionised water. Centrifuge (8000 rpm/min, 10 min) and filter through a 25 μm membrane. Inject the filtrate into a Sephadex S-400 gel column (BV = 50 mL). Elute 1.5 BV using deionised water at a flow rate of 0.6 mL/min, collecting fractions using an automatic fraction collector (3 mL/tube). Trace determination was performed using the anthrone-sulphuric acid method. An elution curve was plotted with the number of effective tubes collected on the x-axis and absorbance at 620 nm on the y-axis. The peak exhibiting the highest polysaccharide content was collected, freeze-dried, and yielded a homogeneous polysaccharide (BRP-3-1).

### 2.3. Chemical Composition Analysis of Polysaccharides

#### 2.3.1. Determination of Polysaccharide Content

Total sugar content of polysaccharide samples was determined using the anthrone-sulfuric acid method [16]: (1) Prepare a 0.2% anthrone-sulfuric acid solution using 98% concentrated sulfuric acid; (2) Accurately prepare a 0.6 mg/mL standard glucose solution and prepare glucose standard solutions according to Table S3. Plot a standard curve using deionized water as the blank, with glucose mass concentration on the x-axis and absorbance on the y-axis. Perform linear fitting on the glucose standard curve, yielding the regression equation: *y* = 2.5688 *x* + 0.1929, R^2^ = 0.9992. (3) Determination of total sugar content: Briefly, 20.00 mg of the polysaccharide sample was accurately weighed, fully dissolved in distilled water, and transferred into a 100 mL volumetric flask to prepare a stock solution with a final concentration of 200 μg/mL. Then, 50 µL of the polysaccharide solution was transferred into a 96-well plate, followed by the addition of 200 µL of anthrone-sulfuric acid solution. The mixture was incubated in the dark for 10 min, and the absorbance at 620 nm was measured using a Multiskan FC microplate reader (Metsys Instruments (Shanghai) Co., Shanghai, Ltd., China). The polysaccharide content was calculated from the absorbance values based on the standard calibration curve, using Equation (1):

Polysaccharide content (%) = (C × V)/m × 100%(1)

where: C is the sample concentration in mg/mL; V is the sample volume adjusted to final volume in mL; m is the precise weight of the polysaccharide sample in mg.

#### 2.3.2. Determination of Protein Residues in Polysaccharides

Bradford assay [17,18] for determining protein content in polysaccharide samples: (1) Weigh 0.01 g of Coomassie Brilliant Blue G250, dissolve in 5 mL ethanol, add 10 mL phosphoric acid, dilute to 100 mL with water, and mix thoroughly. Filter, collect the filtrate to obtain the Coomassie Brilliant Blue solution; (2) Preparation of protein standard curve: Accurately prepare a 0.1 mg/mL bovine serum albumin (BSA) standard solution and proceed according to Table S4. Use deionized water as the blank. Measure the absorbance at 595 nm using a Multiskan FC microplate reader (Merkur Scientific (Shanghai) Instrument Co., Ltd., Shanghai, China). Plot the BSA mass concentration on the *x*-axis and the absorbance on the *y*-axis to perform linear regression. The protein standard linear regression equation is: *y* = 4.4008 *x* + 0.4422, with a linear relationship R^2^ = 0.9967; (3) Protein content determination: A 20.00 mg portion of the polysaccharide sample was accurately weighed, fully dissolved, and diluted to 100 mL in a volumetric flask to prepare a 200 μg/mL sample solution. Then, 50 μL of the solution was transferred into a 96-well plate, followed by the addition of 200 μL Coomassie Brilliant Blue staining solution. After thorough mixing, the absorbance at 595 nm was measured using a Multiskan FC microplate reader (Merck Serono (Shanghai) Instrument Co., Ltd., Shanghai, China). The protein concentration was calculated against the standard calibration curve. Calculate the protein content using Equation (2):

(Protein content (%) = (C × V)/m × 100%(2)

where: C is the concentration of protein content in the sample, in μg/mL; V is the final volume of the sample, in mL; m is the precise weight of the polysaccharide sample, in μg.

#### 2.3.3. Determination of Uronic Acid Contents

Determination of glucuronic acid content in polysaccharide samples using the m-hydroxy biphenyl method [19]: (1) Accurately weigh an appropriate amount of sodium tetraborate, dilute with concentrated sulfuric acid to volume, and prepare a 0.01 mol/L sodium tetraborate-sulfuric acid solution; (2) Accurately weigh an appropriate amount of m-hydroxydiphenyl, dissolve it in 0.5% NaOH solution, and prepare a 0.15% m-hydroxydiphenyl test solution; (3) Accurately weigh D-galacturonic acid (D-Gal) reference standard, dissolve and dilute to volume with distilled water to prepare a 0.1 mg/mL galacturonic acid standard solution. Prepare a series of galacturonic acid standard solutions at various concentrations according to Table S5. Using deionized water as the blank, measure the absorbance at 525 nm using a Multiskan FC microplate reader (Merkur Scientific (Shanghai) Instrument Co., Ltd., Shanghai, China). Perform linear regression with D-Gal mass concentration on the x-axis and absorbance on the y-axis to obtain the galacturonic acid linear equation: *y* = 6.8907 *x* + 0.0728, R^2^ = 0.9974. (4) Determination of galacturonic acid content in samples: Accurately weigh an appropriate amount of polysaccharide sample, dissolve in distilled water, and adjust to a final concentration of 200 µg/mL. Accurately pipette 100 μL of the polysaccharide sample solution into a test tube. Add 600 μL of sodium tetraborate solution, shake well, and heat in a boiling water bath for 12 min. Remove and cool to room temperature. Add 8 μL of m-hydroxy biphenyl solution and allow to react at room temperature for 40 min. Precisely pipette 250 μL of the reaction mixture into a 96-well plate. The absorbance was measured at 525 nm using a Multiskan FC microplate reader (Merck Serendipity (Shanghai) Instrument Co., Ltd., Shanghai, China). Plot the data on the standard curve to determine the glucuronic acid concentration in the polysaccharide solution. Calculate the glucuronic acid content using the following Equation (3):

Uronic acid content= (%) = (C × V)/m × 100%(3)

where: C is the concentration of sugar-aldehyde in the sample, in μg/mL; V is the final volume of the sample, in mL; m is the precise weight of the polysaccharide sample, in μg.

### 2.4. Structural Confirmation of Polysaccharides

#### 2.4.1. Determination of Molecular Weight by High-Performance Gel Permeation Chromatography (HPGPC)

Chromatographic conditions: HPGPC consisted of a Waters 1515 high performance liquid chromatograph (Waters Corporation, Milford, MA, USA), a Waters 2410 Refractive Index Detector (Waters Corporation, USA), and three Shodex Ohpak polymer matrix water-soluble SEC (GFC) columns (SB-803 HQ, SB-804 HQ, and SB-805 HQ) connected in series (8 mm × 300 mm, Nippon Shokubai Electric Works Co., Ltd., Tokyo, Japan). The chromatographic conditions were set as follows: mobile phase, 0.05 M NaCl solution; flow rate, 0.65 mL/min; column temperature, 40 °C; and injection volume, 30 μL.

#### 2.4.2. Determination of Monosaccharide Composition

Hydrolysis of Polysaccharides

Accurately weigh 5 mg of polysaccharide sample and add to 1 mL of 2 mol/L trichloroformic acid (TFA) solution. React at 120 °C for 2 h, then evaporate to dryness. Add 3 mL of methanol, evaporate by rotary evaporation, and repeat the process 2–3 times. Dissolve in 5 mL distilled water. Take 0.2 mL of the polysaccharide hydrolysate, add 0.2 mL 0.5 mol/L NaOH solution and 0.5 mL 0.5 mol/L PMP in methanol. Mix thoroughly and react at 70 °C for 1 h. Add 0.2 mL 0.5 mol/L HCl and extract three times with 1 mL chloroform. Discard the chloroform layers. Take 0.3 mL and dilute to 1 mL with deionised water.

Monosaccharide Assay

A Thermo U3000 liquid chromatography system (Thermo Scientific, Waltham, MA, USA) was used, with a ZORBAX Eclipse XDB-C18 column (Agilent Technologies, Santa Clara, CA, USA). The mobile phase consisted of acetonitrile and phosphate buffer at a volume ratio of 17:83, the flow rate was set to 0.8 mL/min, the column temperature was maintained at 30 °C, the detection wavelength was 250 nm, the injection volume was 10 μL, and ultraviolet spectrophotometry was employed for detection.

#### 2.4.3. Impurity Inspection Based on UV Spectroscopy

A polysaccharide solution (0.1 mg/mL) was prepared with deionized water. UV absorption scanning was performed from 200 to 500 nm using a Mettler Toledo UV5-Bio UV spectrophotometer (Mettler Toledo Technologies (China) Co., Ltd., Shanghai, China), and the UV absorption spectrum of the polysaccharide was recorded.

#### 2.4.4. Fourier Transform Infrared Spectroscopy for Functional Group Identification

The polysaccharide was mixed with potassium bromide powder (1:100), ground and compressed into pellets. FT-IR spectra were recorded using a Shimadzu Prestige-21 Fourier transform infrared (FT-IR) spectrophotometer (Shimadzu Corporation, Kyoto, Japan) over the range of 4000–400 cm^−1^.

#### 2.4.5. Methylation Analysis

Methylation Reaction

Weigh 10 mg of the polysaccharide sample, dissolve in 1 mL ultra-purified water, add 1 mL of 100 mg/mL carbodiimide and react for 2 h. Add 1 mL of 2 M imidazole, followed by 1 mL of 30 mg/mL NaBD_4_, and react for three hours. Add 100 μL of glacial acetic acid to terminate the reaction. Dialyse the sample for 24 h. Following dialysis, freeze-dry the sample and proceed with methylation. Dissolve the freeze-dried sample in 500 μL DMSO. Accurately weigh 1 mg of the test sample and dissolve in 500 μL DMSO. Add 1 mg NaOH and incubate for 30 min. Add 50 μL of methyl iodide solution and react for one hour. Add 1 mL water and 2 mL dichloromethane, vortex mix, centrifuge, and discard the aqueous layer. Repeat the aqueous wash three times. Aspirate the lower dichloromethane layer and evaporate to dryness. One hundred microliters (100 μL) of 2 M trifluoroacetic acid (TFA) was added, and the mixture was incubated at 121 °C for 90 min. The mixture was then evaporated to dryness at 30 °C. Next, 50 μL of 2 M ammonia solution and 50 μL of 1 M sodium borodeuteride (NaBD_4_) were added, mixed thoroughly, and the reaction was allowed to proceed at room temperature for 2.5 h. Twenty microliters (20 μL) of acetic acid was added to terminate the reaction, followed by evaporation to dryness under nitrogen. The residue was washed twice with 250 μL of methanol, and evaporated to dryness under nitrogen after each wash. Subsequently, 250 μL of acetic anhydride was added, and the mixture was vortexed thoroughly to ensure uniform mixing. The mixture was incubated at 100 °C for 2.5 h, after which 1 mL of deionized water was added and allowed to stand for 10 min. Then, 500 μL of dichloromethane was added, the mixture was vortexed for mixing and centrifuged, and the aqueous layer was discarded. The water washing step was repeated three times. Finally, the lower dichloromethane layer was collected for instrumental analysis.

GC-MS Conditions for Methylation Product Detection

Chromatography system: An Agilent 7890A gas chromatography (GC) system (Agilent Technologies, Santa Clara, CA, USA) was employed, equipped with an HP-5MS capillary column (30 m × 0.25 mm × 0.25 μm; Agilent J&W Scientific, Folsom, CA, USA). Helium was used as the carrier gas at a constant flow rate of 1.0 mL/min. The injector temperature was set to 260 °C, the injection volume was 1 μL, and the split ratio was maintained at 10:1 with a solvent delay of 2.2 min. The column temperature program was as follows: initial hold at 50 °C for 1.0 min, followed by a linear ramp of 50 °C/min to 130 °C, then a subsequent ramp of 3 °C/min to 230 °C, and finally a hold at 230 °C for 2 min.

Mass Conditions: A quadrupole mass spectrometry detection system (Agilent 5977B; Agilent Technologies, Santa Clara, CA, USA) was utilized, featuring an Electron Impact (EI) ion source. The inlet temperature was set to 230 °C, and the quadrupole temperature was maintained at 150 °C. The electron energy was 70 eV, with the scan mode set to full scan (SCAN) and a mass scan range (*m*/*z*) of 30–600.

#### 2.4.6. NMR Detection for Structure Elucidation

A solution of BRP-3-1 at a concentration of 10.0 mg/mL was prepared using heavy water as the solvent. The ^1^H NMR, ^13^C NMR, COSY, HSQC, HMBC, and NOESY nuclear magnetic resonance spectra of BRP-3-1 were determined using an AV-400 nuclear magnetic resonance spectrometer (Thermo Fisher, Waltham, MA, USA).

### 2.5. Pharmaceutical Research on Regulating Glucose and Lipid Metabolism

Using the HepG-2 cell insulin resistance model, the effects of BRP-1, BRP-2, and BRP-3-1 on glucose and lipid metabolism in HepG-2 cells were examined, with glucose consumption and total cholesterol (TC) and triglyceride (TG) metabolism serving as indicators.

#### 2.5.1. Cell Culture

HepG2 cells (Chinese Academy of Sciences Cell Bank, Shanghai, China) were cultured in 25 cm^2^ cell culture flasks with high-glucose DMEM supplemented with 10% fetal bovine serum (FBS) and 1% penicillin–streptomycin solution. High-glucose DMEM was procured from Thermo Fisher Scientific (Suzhou, China). Cells were maintained at 37 °C in a humidified incubator with 5% CO_2_ (Model MCO-18AIC, SANYO, Moriguchi, Japan). Cells were passaged at a 1:3 ratio every 2–3 days, and the medium was replaced on the day after passaging.

#### 2.5.2. MTT Assay

Collect HepG-2 cells in the logarithmic growth phase, digest conventionally, centrifuged, counted, and adjusted to a concentration of 1 × 10^5^ cells/mL in DMEM high-glucose medium supplemented with 10% FBS. They were then seeded into a 96-well plate at 100 μL per well, with peripheral wells filled with 150 μL sterile phosphate-buffered saline (PBS) as humidification wells. The plate was incubated for 24 h in a CO_2_ cell culture incubator (Model MCO-18AIC, SANYO, Japan). Dissolve BRP in phosphate-buffered saline and serially dilute with DMEM high-glucose medium containing 10% FBS to the desired concentration. Upon completion of cell culture, the blank group and normal control group were each added 100 μL of cell culture medium. Each experimental group was added the corresponding concentration of test substance to the 96-well plate, with final concentrations of 800, 400, 200, 100, 50, and 25 μg/mL, respectively. Each concentration group comprised eight replicate wells, which were further cultured in the cell incubator. After 24 h, cell growth was observed under a microscope. Subsequently, 20 μL of MTT solution (5 mg/mL, prepared in phosphate-buffered saline) was added, and the plates were incubated in the cell culture incubator for 3 h. Following incubation, discard the cell culture medium from the 96-well plate and add 150 μL of dimethyl sulfoxide (DMSO) solution. Using a GO-1510 microplate reader (Thermo Fisher Scientific, USA), detect and record the optical density (OD) value at a wavelength of 490 nm. The experiment was repeated three times, and the cell survival rate was calculated. Cell survival rate (%) = 100 × (OD experimental group—OD blank group)/(OD normal control group—OD blank well). The MTT assay results are presented in Tables S1–S3.

#### 2.5.3. Establishment of a PA/OA-Induced Metabolic Dysfunction Model in HepG-2 Cells and Pharmacological Intervention

Collect HepG-2 cells in the logarithmic growth phase, digest conventionally, centrifuge, count, and adjust cell concentration to 5 × 10^4^ cells/mL using DMEM high-glucose medium supplemented with 10% FBS. Seed 100 μL per well into a 96-well plate, and fill peripheral wells with 150 μL sterile phosphate-buffered saline (PBS) as moisture wells. Incubate in a CO_2_ cell culture incubator for 24 h. The following day, appropriate quantities of palmitic acid (PA) and oleic acid (OA) were dissolved in water at 75 °C. While hot, these solutions were mixed with 20% BSA (free of fatty acids) dissolved in phosphate-buffered saline (PBS) at pH 7.4, then filtered. Immediately prior to use, dilute with complete medium to prepare fresh culture medium containing 3% BSA, 1 mmol/L PA, and 2 mmol/L OA. For model establishment, discard the supernatant from the 96-well plate. Add high-glucose DMEM medium containing 10% FBS to the normal control wells. Add fresh complete medium containing 3% BSA, 1 mmol/L PA, and 2 mmol/L OA to all other wells and incubate for 24 h to induce insulin resistance in HepG-2 cells.

After model establishment, the wells were divided into the following groups: blank control group, model control group, and high-, medium-, and low-dose groups of the test substance. The supernatants in the blank control and model control groups were discarded and replaced with complete medium; the test substance groups were replaced with complete medium containing the corresponding concentrations of the test substance, followed by continuous incubation for 24 h. The experiment was performed in four independent replicates.

Based on the MTT assay results, three concentrations were selected for BRP-1 and BRP-3-1: 800 (high), 400 (medium), and 200 (low) μg/mL. For BRP-2, the concentrations were set as 100, 50, and 25 μg/mL. Subsequently, 100 μL of the sample solution at each concentration was added to the corresponding wells.

#### 2.5.4. Glucose Consumption Testing

Take the supernatant and, following the method outlined in the glucose oxidase assay kit instructions, Measure The Glucose Concentration in each well using a multi-mode microplate reader. Glucose concentration (mmol/L) = sample dilution factor × C standard × (A measured − A blank)/(A standard − A blank). Glucose consumption = Glucose content in blank control group − Glucose content in test group.

#### 2.5.5. Testing for TC and TG

Take an appropriate amount of logarithmic-phase HepG2 cells and seed at 1.0 × 10^5^ cells per well in a 6-well plate to establish an insulin-resistant HepG2 cell model. Administer drugs in groups for 24 h. Discard the supernatant, wash the cells twice with pre-chilled PBS, add RIPA lysis buffer, mix thoroughly, and allow to stand for 10 min. Collect the cell lysates from each group, heat at 70 °C for 10 min, centrifuge at 2000 rpm for 5 min, and collect the supernatant. Following the protocol specified in the respective assay kit manual, measure the TG and TC levels in each group of cells using a multi-mode microplate reader.

#### 2.5.6. Data Analysis

The data obtained in this study were statistically analyzed using GraphPad Prism 9.0 software. Results are expressed as mean ± standard deviation (Mean ± SD). After passing normality and homogeneity of variance tests, one-way analysis of variance (one-way ANOVA) was performed to evaluate overall intergroup differences, followed by Dunnett’s multiple comparisons test to compare each BRP treatment group with the model group and correct for multiple testing errors at a significance level of α = 0.05. Statistical significance is indicated by *p* < 0.05, * *p* < 0.01, ** *p* < 0.001, and ** *p* < 0.0001 versus the model group, with *p* < 0.05 considered statistically significant.

## 3. Results and Discussion

### 3.1. Preparation and Purification of Polysaccharides

**Figure 1:**
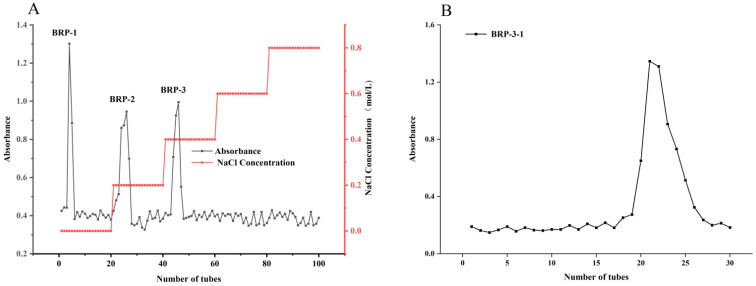
DEAE-FF elution curve of BRP. DEAE-FF elution curve of BRPs (**A**); sephacryl S-400 elution curve of BRP-3-1 (**B**).

Rough *B. rapa* L. polysaccharides (BRP) were obtained from the decoction extract of *B. rapa* L. via dialysis (molecular weight cut-off 8000–14000 Da). Subsequently, separation was performed using DEAE-FF an ion exchange resin, followed by sequential elution with 0.2 mol/L and 0.4 mol/L NaCl solutions. The elution curve of absorbance versus elution tube number was plotted using the anthrone-sulfuric acid colorimetric method (Figure 1A). Based on the chromatographic peaks observed in the elution curve, three distinct polysaccharide fractions were collected and designated as BRP-1, BRP-2, and BRP-3. Further purification of the BRP-3 fraction was performed using a Sephadex S-400 gel column. The anthrone-sulfuric acid detection elution curve is shown in Figure 1B, ultimately yielding the homogeneous polysaccharide fraction BRP-3-1.

### 3.2. Chemical Composition Analysis and Impurity Testing of Crude Polysaccharides

#### 3.2.1. Macroelement Analysis of Polysaccharide Chemical Composition

Weigh the freeze-dried powders of each polysaccharide fractions: BRP, BRP-1, BRP-2, and BRP-3. Calculate the yield rate of each component (polysaccharide freeze-dried powder mass × 100/raw material mass). The sugar content in crude polysaccharides, residual protein levels, and uronic acid content in polysaccharides were determined using the anthrone-sulfuric acid method, Coomassie Brilliant Blue method, and sodium tetraborate method, respectively. Results are shown in Table 1.

Results indicate: BRP-1 exhibits the highest sugar content (93.29%); BRP-3 contains the highest uronic acid content (76.64%), demonstrating distinct acidic polysaccharide structural characteristics. Protein residue testing confirms that BRP-1, BRP-2, and BRP-3 contain no detectable protein residues.

#### 3.2.2. Impurity Detection Based on Ultraviolet (UV) Spectroscopy

**Figure 2:**
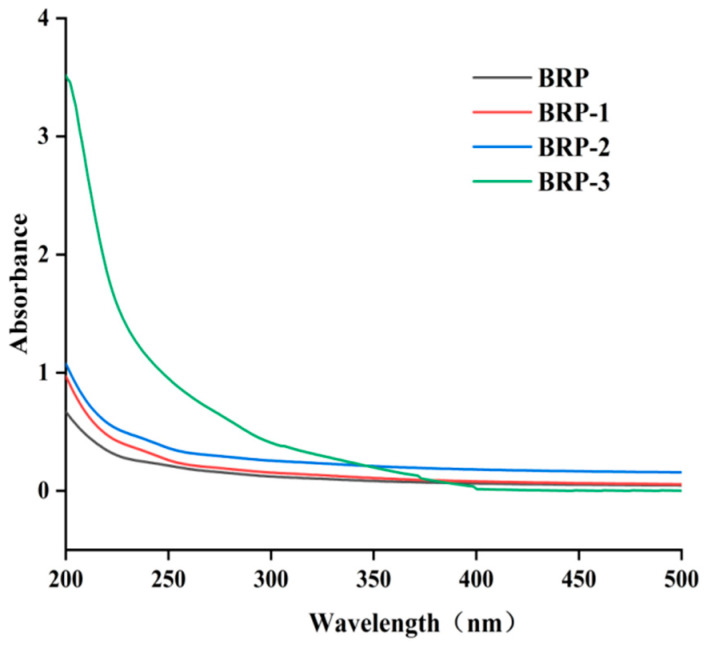
Ultraviolet absorption spectra of BRPs.

The UV spectra of BRP, BRP-1, BRP-2, and BRP-3 are shown in Figure 2. As wavelength increases, the absorbance of the polysaccharides gradually decreases, consistent with the characteristics of most plant polysaccharides. No UV absorption peaks were detected at 260, 280, and 520 nm, indicating that BRP-1, BRP-2, and BRP-3 contain negligible amounts of nucleic acids, proteins, and pigments.

### 3.3. Morphological Characterization of Polysaccharides

In terms of macroscopic morphology, the sample color gradually deepened from the white of BRP-1 to the pale yellow of BRP-2 and the yellow of BRP-3. The appearance changed from the fluffy flocculent structure of BRP-1 to the granular structure of BRP-2 and the flake-like structure of BRP-3.

**Figure 3:**
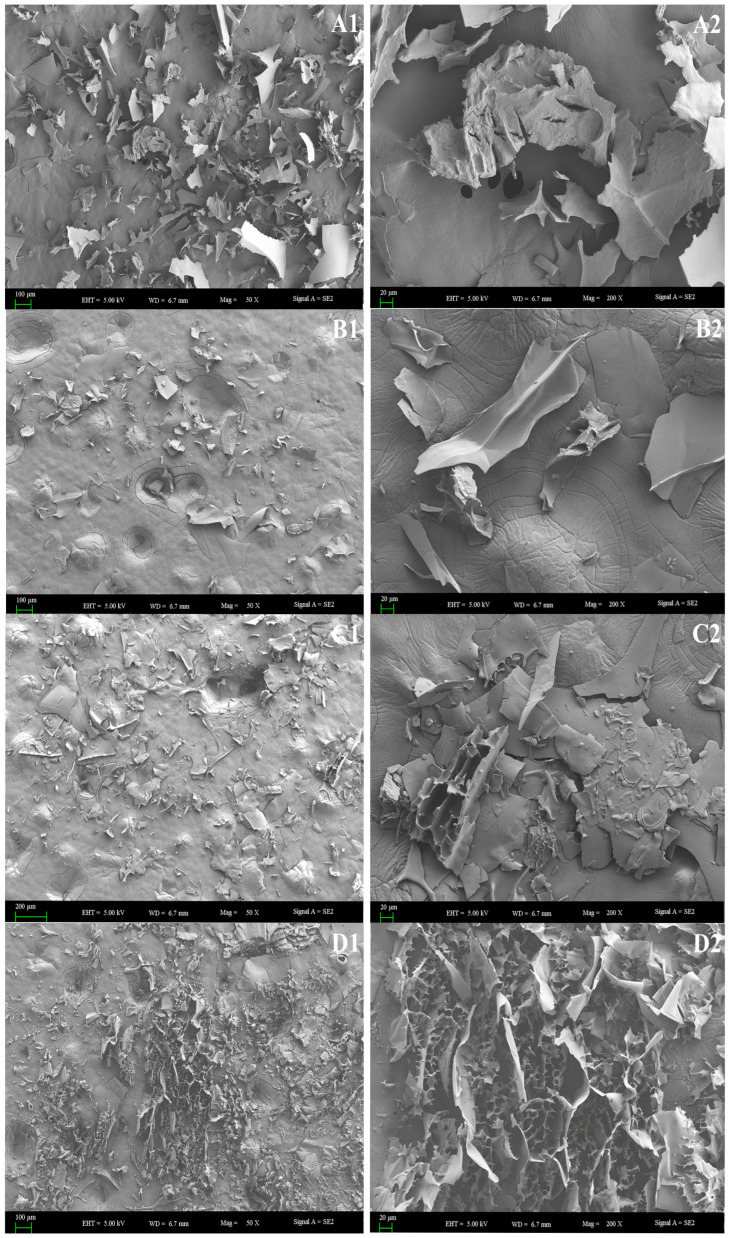
The morphology of BRP under a scanning electron microscope. Representative micrographs of BRP-1 (**A**), BRP-2 (**B**), BRP-3 (**C**), and BRP-3-1 (**D**). Images were captured at low (100×; **A1**,**B1**,**C1**,**D1**) and high (500×; **A2**,**B2**,**C2**,**D2**) magnifications.

The texture and surface microstructure of the polysaccharide samples were examined using a Zeiss Supra 55 VP scanning electron microscope (Carl Zeiss (Shanghai) Management Co., Ltd., Shanghai, China). Results revealed that BRP-1 exhibited a fragmented structure with numerous surface wrinkles and cracks, distributed unevenly (Figure 3(A1,A2)); BRP-2 appeared generally smoother but contained numerous irregular depressions and fragmented areas with varying depths and lengths (Figure 3(B1)). High-magnification observation revealed distinct wrinkles, indicating strong self-assembly capabilities of the polysaccharide molecules (Figure 3(B2)); BRP-3 exhibits droplet-like protrusions and lamellar arrangements (Figure 3(C1)). Its overall morphology reflects the formation of a complex three-dimensional network structure during drying, endowing it with a high specific surface area (Figure 3(C2)). This structure enhances the polysaccharide’s solubility. BRP-3-1 exhibits a typical porous sponge-like morphology with pore sizes uniformly distributed between 100–500 nm. The pore walls are thin and continuous, with no discernible granular or fibrous structures (Figure 3(D1,D2)).

These structural features, particularly the significantly increased specific surface area, provide a crucial morphological foundation for enhancing the polysaccharide’s functional properties in adsorption, water retention, thickening, encapsulation, and reactivity.

### 3.4. Structure Characterisation

#### 3.4.1. Determination of Molecular Weight

**Figure 4:**
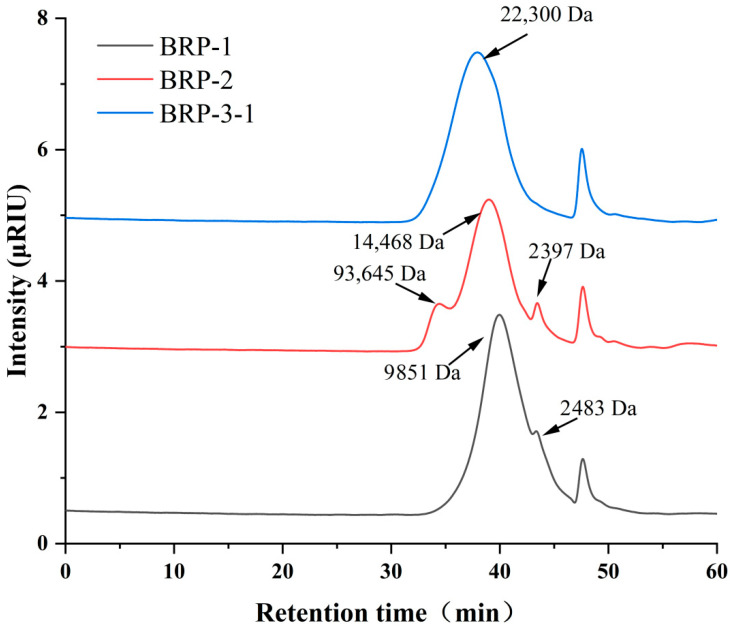
Molecular weight distribution profile of BRPs.

Molecular weight was determined by high-performance gel permeation chromatography (HPGPC) based on the retention time of polysaccharides. The molecular weight determination curve is shown in Figure 4; the molecular weight distribution of BRP-1, BRP-2, and BRP-3-1 is presented in Table 2.

#### 3.4.2. Composition of Monosaccharides

**Figure 5:**
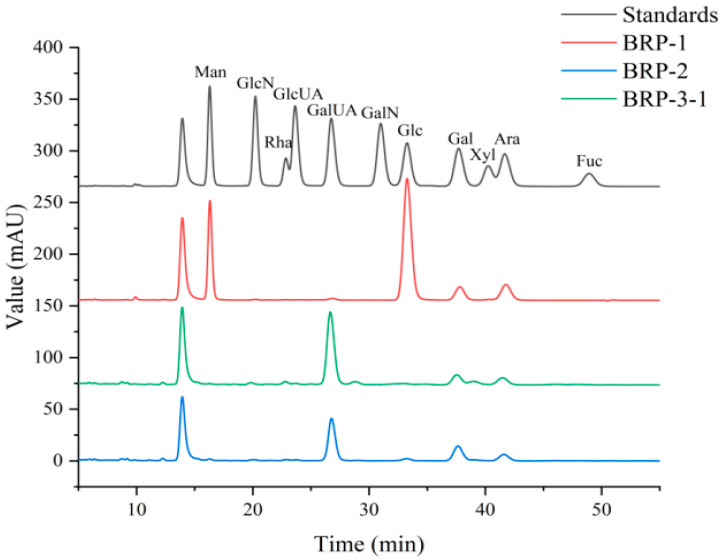
Chromatograms of monosaccharide composition in BRP fractions.

The monosaccharide composition of BRP-1, BRP-2, and BRP-3-1 was analyzed using 1-phenyl-3-methyl-5-pyrazolone (PMP) derivatization. The distribution of acid hydrolysis products on the chromatogram is shown in Figure 5, with the specific composition and molar percentages of each monosaccharide listed in Table 3. The results indicate that BRP-1, BRP-2, and BRP-3-1 all comprise mannose (Man), rhamnose (Rha), galacturonic acid (GalA), glucose (Glc), galactose (Gal), and arabinose (Ara), suggesting that all three are heteropolysaccharides. The monosaccharide BRP-3-1 consists of Rha, Gal, Ara, and GalA, with GalA accounting for a high molar percentage of 75.584%, exhibiting strong characteristics of an acidic polysaccharide structure.

#### 3.4.3. Functional Group Identification Based on Infrared (IR) Spectroscopy

**Figure 6:**
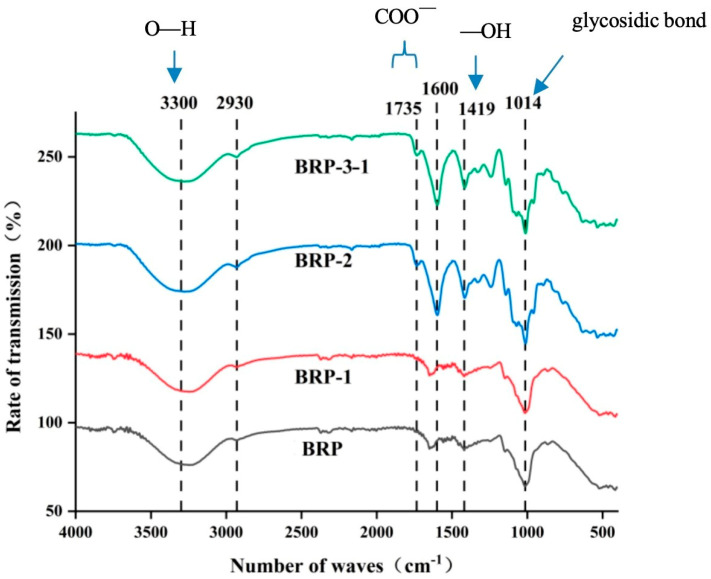
Infrared spectra of BRPs.

The IR spectra of BRP, BRP-1, BRP-2, and BRP-3-1 are shown in Figure 6. The positions of the main absorption peaks are essentially consistent across all samples, indicating shared structural characteristics. A broad, intense absorption peak near 3300 cm^−1^ is attributed to O-H stretching vibrations, suggesting the presence of intermolecular or intramolecular hydrogen bonds within the polysaccharide molecules. Extensive hydrogen bonding of hydroxyl groups causes significant broadening of this peak. The absorption peak at 2930 cm^−1^ corresponds to C-H stretching vibrations, a typical characteristic of carbohydrates. Absorption at 1735 cm^−1^ and 1600 cm^−1^ indicates the presence of -COO^−^ groups in the samples. The absorption at 1419 cm^−1^ is attributed to O-H bending vibrations, further confirming the presence of hydroxyl groups in all four polysaccharides. A sharp, strong absorption peak near 1014 cm^−1^ corresponds to the typical vibrational pattern of glycosidic bonds in polysaccharides [20].

#### 3.4.4. Research on Glycosyl Residues Based on Methylation Reactions

**Figure 7:**
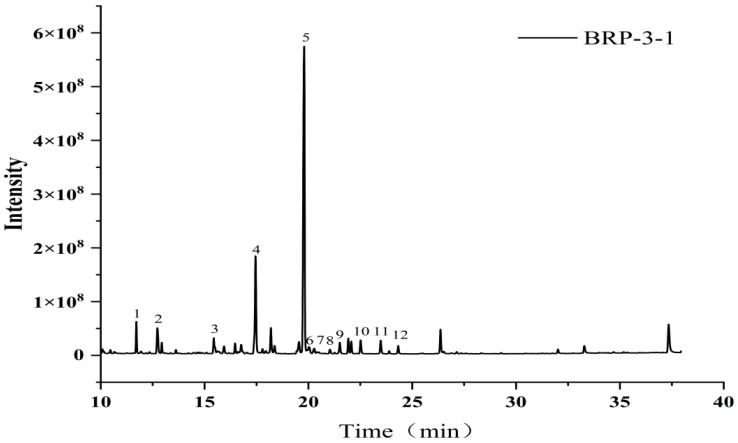
GC-MS total ion chromatogram of BRP-3-1 methylation product.

Following methylation of BRP-3-1, the product was analyzed via GC-MS (Figure 7). Mass spectrometry identification data (Table 4) indicate that BRP-3-1 primarily consists of T-Araf (4.366%), T-Rhap (5.456%), →1,5-Araf (3.109%), T-Galp (3.273%), T-GalpA (14.078%), and →1,4-GalpA (58.450%). The predominant terminal residues are T-Araf and T-Rhap, while the backbone structure is primarily composed of →4)-GalpA-(1→ and →4)-GalpAMe-(1→ units. Key linkage sites include O-1 and O-4. This structural information is consistent with the monosaccharide composition analysis of BRP-3-1.

#### 3.4.5. Determination of Polysaccharide Structures Based on NMR

**Figure 8:**
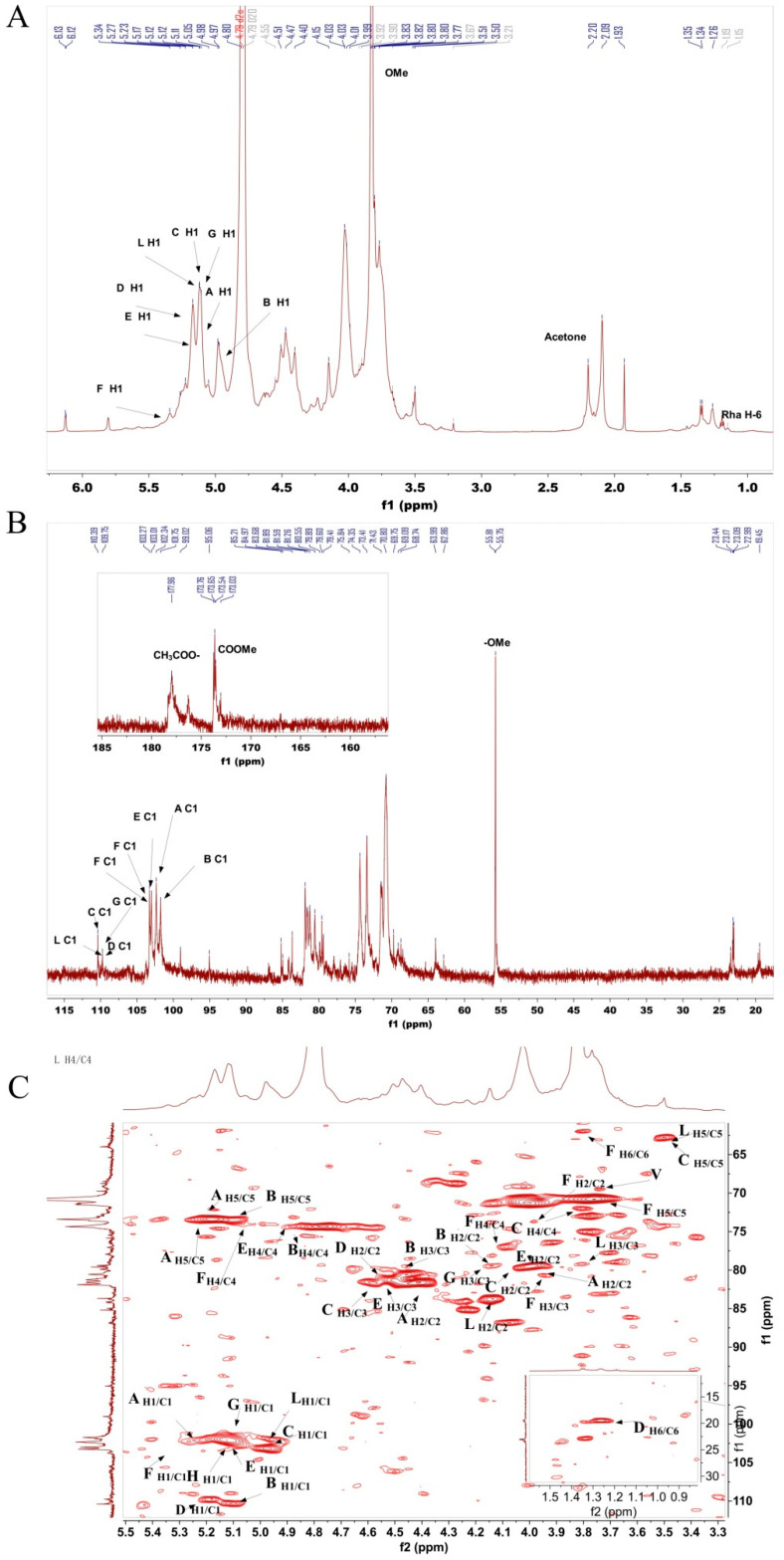

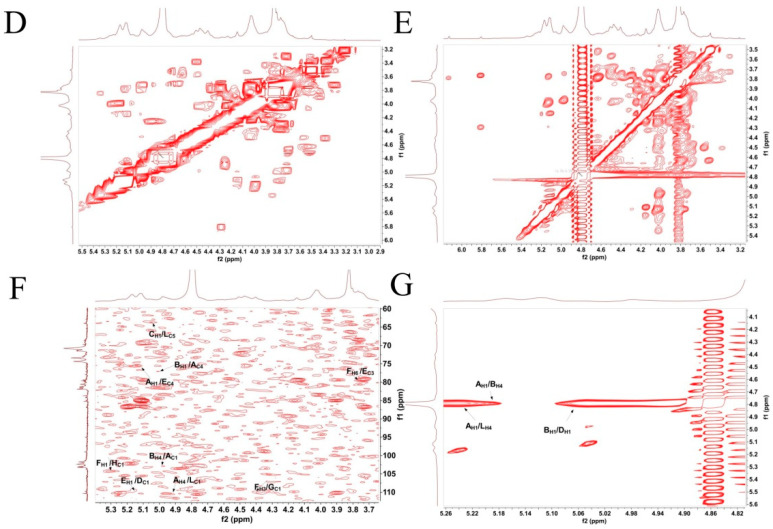
NMR spectra of BRP-3-1. ^1^H-NMR (**A**); ^13^C-NMR (**B**); HMQC (**C**); COSY (**D**); TOCSY (**E**); HMBC (**F**); NOESY (**G**).

The ^1^H-NMR spectrum of BRP-3-1 (Figure 8A) shows a strong peak at δ 4.79 ppm corresponding to the D_2_O solvent peak. The range δ 3.4–5.3 ppm indicates the presence of multiple sugar residues in the structure. Within the δ 3.5–4.4 ppm range, hydrogen signals are severely overlapping, corresponding to protons H2 to H6 on the sugar ring. Multiple signals observed in the terminal proton region (4.45–5.85 ppm) were assigned to the terminal protons of α-GalpA, α-GalpAEt, T-Ara, T-Rha, 1,3,4-GalpA, 1,3,6-Galp, T-GalpA, and T-Galp. The terminal hydrogen signals were assigned to δ 5.12, 4.97, 5.11, 5.17, 5.17, 5.33, 5.12, and 5.27 ppm, respectively. Terminal proton signals cluster around δ 5.11 ppm, indicating the presence of an α-configuration sugar ring structure in BRP-3-1. Additionally, δ 1.19 ppm corresponds to the methyl hydrogen (H6) of the Rha residue, while δ 3.87 ppm corresponds to the proton of the methoxy group (–OCH_3_) in α-GalpAMe [21].

In the ^13^C-NMR spectrum (Figure 8B), BRP-3-1 exhibited multiple anomeric carbon signals in the range of δ 90–110 ppm, which were predominantly distributed around δ 101 ppm. Combined with the chemical shifts of anomeric hydrogen signals in the ^1^H-NMR spectrum, the α-configuration of the sugar moieties was further confirmed. Signals in the range of δ 62–82 ppm showed significant overlap, which was mainly derived from the resonances of C2–C6 of the sugar units. The signal at δ 173.65 ppm was assigned to the carbonyl carbon of uronic acid, indicating the presence of uronic acid structures in BRP-3-1. This result was consistent with the carboxyl absorption peak in the infrared spectrum and the conclusion that BRP-3-1 is an acidic polysaccharide [22,23]. The signal at δ 19.45 ppm corresponded to the C6 (–CH_3_) of rhamnose (Rha) residues, and the signal at δ 52.96 ppm was attributed to the methoxy carbon. The anomeric carbon chemical shifts of α-GalpA, α-GalpAMe, T-Araf, T-Rha, 1,3,4-GalpA, 1,3,6-Galp, T-GalpA and T-Galp residues were δ 102.40, 101.75, 110.39, 109.75, 103.10, 103.27, 109.75 and 103.1 ppm, respectively.

Furthermore, the HSQC spectrum (Figure 8C) displayed the following key C–H correlation signals: δ 5.12/102.4, 4.97/101.75, 5.11/110.39, 5.17/98.91, 5.17/103.1, 5.33/103.27, 5.12/109.75, 5.27/103.1, etc., which corresponded to the anomeric C1/H1 of each sugar residue, respectively. The cross peak at δ 3.87/52.96 ppm was assigned to the hydrogen/carbon signals of the methoxy group (–OCH_3_), demonstrating the presence of some α-GalpA residues in the methyl-esterified form. The correlation peak at δ 5.12/102.4 ppm was attributed to the C1/H1 of →4)-α-D-GalpA-(1→ and →4)-α-D-GalpAMe-(1→ [15]. The T-Araf sugar residue showed its characteristic H1/C1 signal at δ 5.11/110.39 ppm. Combined with the detection results of HSQC and COSY (Figure 8C,D), the chemical shifts of its H2/C2, H3/C3, H4/C4 and H5/C5 were assigned as δ 4.47/81.59, 4.03/79.41, 3.71/71.43 and 3.51/62.86 in sequence. Residue D (T-Rha) was identified by the H6/C6 signal at δ 1.19/19.45 ppm; the correlation signal at δ 3.62 ppm in the COSY spectrum was assigned to H5, and its C5 chemical shift was further determined to be δ 70.8 ppm by the TOCSY spectrum (Figure 8E). By analogy, its H4/C4, H3/C3, H2/C2 and H1/C1 were assigned as δ 4.03/79.89, 4.15/83.68, 4.51/81.89 and 5.17/109.75, respectively.

Sugar residue E (1,3,4-GalpA) was identified by the uronic acid carbonyl carbon signal at C6 (δ 173.65 ppm). Combined with the HSQC and COSY spectra, the chemical shifts of its H5/C5, H4/C4, H3/C3, H2/C2 and H1/C1 were assigned as δ 5.11/74.45, 4.97/75.85, 4.40/80.55, 4.01/79.41 and 5.17/103.1 in sequence. Sugar residue F (1,3,6-Galp) exhibited its characteristic anomeric H1/C1 signal at δ 5.33/103.1 ppm; combined with the HSQC and COSY spectra (Figure 8D), the chemical shifts of its H2/C2, H3/C3, H4/C4, H5/C5 and H6/C6 were assigned as δ 4.03/73.41, 4.40/73.41, 4.11/79.41, 4.40/75.84 and 3.76/62.86 in sequence. Sugar residue G (T-GalpA) was identified by the uronic acid carbonyl carbon signal at C6 (δ 173.76 ppm). Combined with the HSQC and COSY spectra, the chemical shifts of its H5/C5, H4/C4, H3/C3, H2/C2 and H1/C1 were assigned as δ 5.11/73.01, 5.05/74.35, 4.15/77.60, 4.02/79.79 and 5.12/109.75 in sequence. Sugar residue H exhibited its characteristic anomeric H1/C1 signal at δ 5.27/103.1 ppm; combined with the HSQC and COSY spectra (Figure 8D), the chemical shifts of its H2/C2, H3/C3, H4/C4, H5/C5 and H6/C6 were assigned as δ 4.03/73.41, 4.47/75.84, 4.15/70.18, 3.76/71.54 and 3.50/63.99 in sequence.

Analysis of the HMBC spectrum (Figure 8F) revealed that the cross peak at δ 5.11/177.96 ppm corresponded to the H5/C6 signal of residue B (→4)-α-D-GalpA-(1→); combined with the HSQC spectrum, the chemical shifts of its H2/C2, H3/C3, H4/C4 and H5/C5 were assigned as δ 4.15/79.41, 4.15/79.41, 4.40/81.26 and 5.11/73.41 in sequence. Similarly, in the HMBC spectrum of residue A (→4)-α-D-GalpA-(1→), the correlation peak at δ 5.17/173.65 ppm was assigned to the H5/C6 signal, and the chemical shifts of its H2/C2, H3/C3, H4/C4 and H5/C5 were assigned as δ 4.03/79.60, 4.14/81.59, 4.98/76.03 and 5.17/74.45 in sequence.

The linkage relationships between sugar residues were further deduced from the HMBC spectrum: the cross peak at δ 4.98/110.39 ppm indicated that C1 of sugar residue C was correlated with H4 of sugar residue A; that at δ 5.12/75.84 ppm indicated that H1 of sugar residue A was correlated with H4 of sugar residue B; and that at δ 4.97/76.03 ppm indicated that H1 of sugar residue B was correlated with C4 of sugar residue A. In the NOESY spectrum (Figure 8F), correlations of H1 of residue A with H4 of residue B, H1 of residue A with H1 of residue C, and H1 of residue B with H4 of residue A were observed, which further confirmed the presence of the linkage sequence →4)-α-D-GalpA-(1→4)-α-D-GalpAMe-(1→4)-α-D-GalpA-(1→ in BRP-3-1. The assignments of chemical shifts for all sugar residues are summarized in Table 5.

The cross peak at δ 5.12/75.85 ppm in the HMBC spectrum indicated that H1 of Residue A was correlated with C4 of Residue E, demonstrating that the α-D-GalpA residue was linked to →3,4)-β-D-GalpA-(1→ via the O-1 bond, namely: →4)-α-D-GalpA-(1→3,4)-β-D-GalpA-(1→. The cross peak at δ 5.17/109.75 ppm indicated that H1 of residue E was correlated with C1 of residue D, demonstrating that the →3,4)-β-D-GalpA-(1→ residue was linked to T-Rha via the O-1 bond, namely: →3,4)-β-D-GalpA-(1→T-Rha. This backbone was designated as side chain of BRP-3-1.

**Figure 9:**
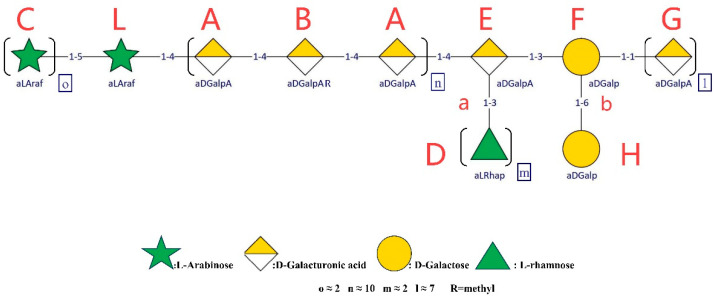
Predicted structure of BRP-3-1.

The cross peak at δ 3.76/80.55 ppm in the HMBC spectrum indicated that H6 of residue F was correlated with C3 of residue E, demonstrating that the α-D-GalpA residue was linked to →3,6)-β-D-Galp-(1→ via the O-3 bond, namely: →4)-α-D-GalpA-(1→3,6)-β-D-Galp-(1→. The cross peak at δ 5.33/103.1 ppm indicated that H1 of residue F was correlated with C1 of residue H, demonstrating that the →3,6)-β-D-Galp-(1→ residue was linked to T-Galp via the O-1 bond, namely: →3,6)-β-D-Galp-(1→1)-β-D-Galp. The cross peak at δ 4.40/109.5 ppm indicated that H3 of residue F was correlated with C1 of residue G, demonstrating that the →3,6)-β-D-Galp-(1→ residue was linked to T-GalpA via the O-3 bond, namely: →1,6)-β-D-Galp-(3→1)-β-D-GalpA. The cross peak at δ 5.33/103.1 ppm indicated that H1 of residue F was correlated with C1 of residue H, demonstrating that the →3,6)-β-D-Galp-(1→ residue was linked to T-Galp via the O-1 bond, namely: →3,6)-β-D-Galp-(1→1)-β-D-Galp. This backbone was designated as side chain b of BRP-3-1. The chemical shift assignments of the sugar residues in the linkage sequences are summarized in Table 5. The structure of BRP-3-1 is shown in Figure 9.

### 3.5. Regulatory Effects of Polysaccharides on Glycolipid Metabolism

#### 3.5.1. Effects of Insulin Resistance on Glucose Uptake in HepG-2 Cells

**Figure 10:**
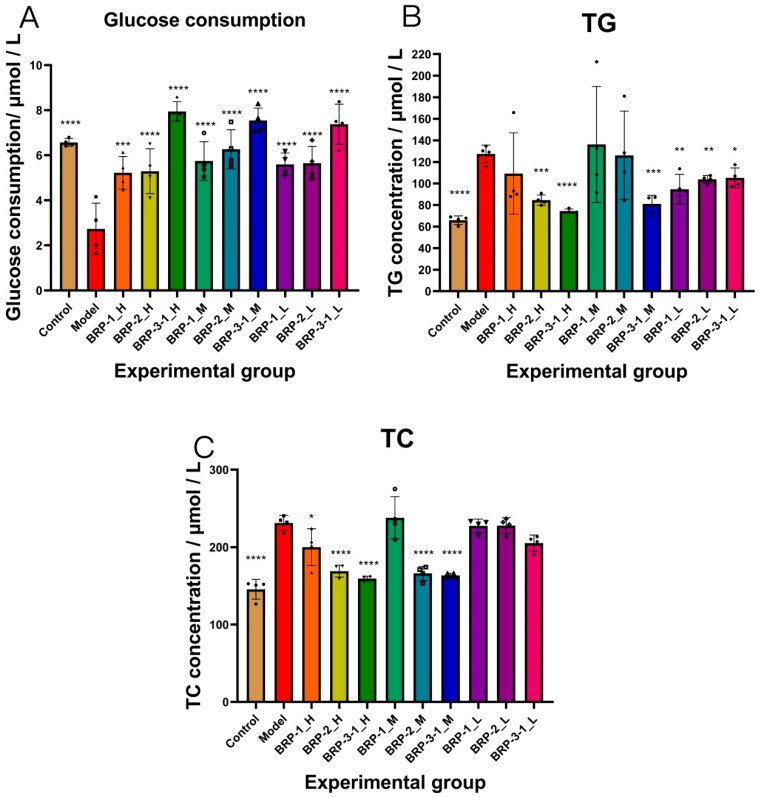
BRP-3-1 Ameliorates glucose and lipid metabolism in PA/OA-induced metabolic dysfunction HepG-2 cells. Glucose consumption of HepG-2 cells in each experimental group (**A**); TG level of HepG-2 cells in each experimental group (**B**); TC level of HepG-2 cells in each experimental group (**C**). Note: * *p* < 0.05, ** *p* < 0.01, *** *p* < 0.001, ****p < 0.0001.

Compared with the model control group, the medium-dose BRP-2 group (50 μg/mL) and the high- and medium-dose BRP-3-1 groups (800 μg/mL and 400 μg/mL, respectively) significantly increased glucose consumption in HepG-2 cells (*p* < 0.05 or *p* < 0.01). Detailed results are presented in Table S4, and comparisons of glucose consumption among groups are shown in Figure 10A.

#### 3.5.2. Insulin Resistance Effects on TC Levels and TG Consumption in HepG-2 Cells

Compared with the model control group, both the high-dose BRP-2 group (100 μg/mL) and the medium-dose group (50 μg/mL), and the high-dose BRP-3-1 group (800 μg/mL) and medium-dose group (400 μg/mL) significantly reduced total cholesterol (TC) levels in insulin-resistant model HepG-2 cells. Additionally, the high-dose BRP-3-1 group (800 μg/mL) significantly reduced triglyceride (TG) levels in cells (Figure 10B,C). While other test compounds showed a trend toward reducing cellular TC and TG levels, these reductions were not statistically significant compared to the model control group did. Detailed measurement results are presented in Table S5.

Glucose consumption analysis (Figure 10A) revealed that high-, medium-, and low-dose BRP-3-1 significantly increased glucose consumption in HepG2 cells compared with the insulin-resistant model group, suggesting that BRP31 effectively improves glucose metabolism disorders in insulin-resistant cells.

Regarding lipid metabolism (Table S5), compared with the normal control group, the model control group exhibited significantly elevated levels of total cholesterol (TC) and triglycerides (TG) in HepG2 cells, confirming the successful establishment of the insulin-resistant model. Compared with the model group, treatment with both BRP2 and BRP-3-1 significantly reduced TC levels in insulin-resistant HepG2 cells. In addition, BRP-3-1 significantly decreased TG levels in model cells (Figure 10B,C).

Glucose consumption, TC, and TG levels are classic indicators for evaluating the efficacy of interventions against insulin resistance. The above in vitro cellular results preliminarily indicate that BRP-3-1 and BRP-2 exert biological activities in regulating glucose and lipid metabolism at the cellular level, thereby improving impaired glucose utilization and lipid accumulation induced by insulin resistance.

However, this study has certain limitations. First, a positive drug control group was not included in this study. Therefore, although the ameliorative effects of the polysaccharides were confirmed, their comparative efficacy with clinically used drugs remains to be further evaluated. Second, HepG2 is a hepatocellular carcinoma cell line, and its metabolic characteristics may differ from those of normal hepatocytes. Third, in vitro models cannot fully simulate the complex in vivo metabolic environment or long-term accumulation toxicity.

Taken together, although the edible safety of *Brassica rapa* L. as a common vegetable has been verified, future studies should include safety evaluations in normal hepatocytes, introduce positive drug controls for efficacy comparison, and further validate long-term feeding toxicity and pharmacodynamic effects using appropriate animal model.

## 4. Conclusions

Acidic polysaccharides possess rich pharmacological activities and, compared to neutral polysaccharides, demonstrate significant advantages in water solubility, solubilising capacity, and swelling properties, making them a current research focus in the field of polysaccharides. This study successfully isolated three *B. rapa* L. root acidic polysaccharide fractions (BRP-1, BRP-2, BRP-3) through a combination of dialysis retention and ion-exchange column chromatography. Further purification via gel-column chromatography yielded the monomeric polysaccharide fraction BRP-3-1, which underwent systematic structural characterisation.

To investigate the biological activity of the isolated acidic polysaccharides, a PA/OA-induced metabolic dysfunction model was conducted using HepG2 cells. The results showed that BRP-2 and BRP-3-1 exert a certain regulatory effect on glycolipid metabolism-related indicators in insulin-resistant HepG2 cells, with specific manifestations as follows:

Enhanced glucose consumption: compared to the model group, the medium-dose BRP-2 group (50 μg/mL), high-dose BRP-3-1 group (800 μg/mL), and medium-dose BRP-3-1 group (400 μg/mL) significantly increased glucose consumption in insulin-resistant HepG2 cells.

Reduced lipid content: The high-dose BRP-2 group (100 μg/mL) and medium-dose group (50 μg/mL), BRP-3-1 high-dose group (800 μg/mL) and medium-dose group (400 μg/mL) significantly reduced total cholesterol (TC) levels in insulin-resistant HepG2 cells; Furthermore, the high-dose BRP-3-1 group (800 μg/mL) also significantly reduced triglyceride (TG) levels in these model cells.

Traditionally, *B. rapa* L. has been recognized for its efficacy in regulating blood glucose and improving blood lipid levels. Through in vitro cell experiments, this study preliminarily verified that its acidic polysaccharide fractions (especially BRP-3-1) can enhance glucose consumption and reduce total cholesterol (TC) as well as triglyceride (TG) levels in insulin-resistant HepG2 cells. These results provide a basic experimental reference for further in-depth investigation of the biological activity mechanisms of *B. rapa* polysaccharides, the conduct of in vivo experimental verification, and the development of related applications.

As a common edible vegetable, the edible safety of *B. rapa* L. rhizome has been verified through long-term practice. This study demonstrated using the HepG2 cell model that the purified homogenous polysaccharide BRP-3-3-1, isolated from *B. rapa* L. rhizome, exhibits the following effects at different doses: increasing glucose consumption in HepG2 cells and reducing intracellular triglyceride (TG) and total cholesterol (TC) levels. These findings suggest that BRP-3-1 exhibits promising in vitro regulatory activity for glucose and lipid metabolism, demonstrating potential value for further development as a functional food ingredient.

In this study, the acidic polysaccharides exhibited a certain ameliorative effect on glucolipid metabolic disorders induced by PA-OA in HepG2 cells. Combined with reported literature [24,25], it is speculated that this biological activity is closely related to its specific structural characteristics. The relatively high uronic acid content and the resulting negative charge on the molecular surface (COO^−^), moderate molecular weight distribution, and high degree of branching may synergistically enhance the water solubility and cell affinity of the polysaccharides, thereby potentially regulating signaling pathways associated with glucolipid metabolism.

Given that the molecular mechanism has not been further explored in this study, the above structure-activity relationship analysis is mainly based on theoretical deduction from existing literature, and the relevant hypotheses still need to be further verified and improved by follow-up experimental data.

## Figures and Tables

**Table 1:** Yield rate and chemical compositions of BRPs.

	BRP-1	BRP-2	BRP-3
Yield rate (wt %, dry sample)	0.66	0.19	1.07
Polysaccharide content (wt %)	93.29	29.18	28.05
Protein content (wt %)	0.00	0.00	0.00
Galacturonic acid content (wt %)	13.05	57.37	76.64

**Table 2:** Molecular weight distribution of BRP-1, BRP-2, BRP-3-1.

Number	RT (min)	*M*_p_ (Da)	*M*_w_ (Da)	*M*_n_ (Da)
BRP-1	39.957	8401	9851	7036
	43.356	2259	2483	1898
BRP-2	34.408	71,687	93,465	59,731
	39.009	12,117	14,468	10,139
	43.443	2185	2397	1835
BRP-3-1	37.942	18,299	22,300	15,297

Note: *M*p: peak molecular weight; *M*w: weight-average molecular weight; *M*n: number-average molecular weight.

**Table 3:** Monosaccharide composition in BRPs Unit: molar %.

Polysaccharide	Man	Rha	GalA	Glc	Gal	Ara
BRP-1	20.494	0.166	0.487	65.123	7.165	6.565
BRP-2	0.416	1.434	55.004	1.370	32.945	8.277
BRP-3-1	-	1.687	75.584	-	14.452	8.277

Note: Man: mannose, Rha: rhamnose, GalA: galacturonic acid, Glc: glucose, Gal: galactose, Ara: arabinose.

**Table 4:** Glycosyl residues of BRP-3-1 based on methylation analysis.

No.	Rt /min	Connection	Derivative Name	Mw	Relative Molar Ratio (%)	Mass-to-Charge Ratio *m*/*z*
1	11.71	T-Ara*f*	1,4-di-*O*-acetyl-2,3,5-tri-*O*-methyl arabinitol	279	4.366	59, 71, 87, 102, 118, 129, 145, 161
2	12.73	T-Rha*p*	1,5-di-*O*-acetyl-6-deoxy-2,3,4-tri-*O*-methyl rhamnitol	293	5.456	59, 85, 102, 118, 131, 145, 162, 189
3	15.45	1,5-Ara*f*	1,4,5-tri-O-acetyl-2,3-di-O-methyl arabinitol	307	3.109	59, 71, 87, 102, 118, 129, 145, 162, 189
4	17.45	T-Gal*p*A	1,5-di-*O*-acetyl-2,3,4,6-tetra-*O*-methyl galactitol	325	17.351	59, 73, 89, 102, 118, 131, 147, 163
5	19.79	1,4-Gal*p*A	1,4,5-tri-*O*-acetyl-2,3,6-tri-*O*-methyl galactitol	353	58.450	59, 75, 87, 102, 118, 129, 144, 162, 175, 205
6	20.03	1,2,3,5-Ara*f*	1,2,3,4,5-penta-*O*-acetyl arabinitol	363	1.463	60, 75, 87, 99, 118, 129, 143, 160, 173, 201, 233
7	20.28	1,3-Gal*p*	1,3,5-tri-*O*-acetyl-2,4,6-tri-*O*-methyl galactitol	351	0.986	60, 71, 87, 101, 118, 129, 143, 161, 174, 189, 233, 245
8	21.51	1,6-Gal*p*	1,5,6-tri-*O*-acetyl-2,3,4-tri-*O*-methyl galactitol	351	1.805	59, 71, 87, 99, 118, 129, 143, 162, 173, 189, 233
9	21.92	1,3,4-Gal*p*A	1,3,4,5-tetra-*O*-acetyl-2,6-di-*O*-methyl galactitol	381	1.960	59, 74, 87, 99, 118, 131, 145, 166, 172, 185, 205, 233
10	22.51	1,2,4-Gal*p*A	1,2,4,5-tetra-*O*-acetyl-3,6-di-*O*-methyl galactitol	381	1.937	60, 72, 88, 101, 115, 130, 140, 160, 175, 190, 235, 249, 305, 361
11	23.48	1,4,6-Gal*p*A	1,4,5,6-tetra-*O*-acetyl-2,3-di-*O*-methyl galactitol	381	1.961	59, 74, 87, 102, 118, 129, 144, 161, 173, 189, 203, 263
12	24.32	1,3,6-Gal*p*	1,3,5,6-tetra-*O*-acetyl-2,4-di-*O*-methyl galactitol	379	1.156	59, 74, 87, 101, 118, 129, 139, 160, 174, 189, 202, 234, 245, 305

**Table 5:** Major glycosyl residues in BRP-3-1 identified by methylation analysis.

Glycosyl Residue		Chemical Displacement/ppm					
		H1/C1	H2/C2	H3/C3	H4/C4	H5/C5	H6/C6
A	→4)-α-Gal*p*A-(1	5.12/102.4	4.03/79.6	4.14/81.59	4.98/76.03	5.17/74.45	173.65
B	→4)-α-Gal*p*AEt-(1→	4.97/101.75	4.15/79.41	4.4/81.26	4.97/75.84	5.11/73.41	177.96
C	T-Ara*f*	5.11/110.39	4.47/81.59	4.03/79.41	3.71/71.43	3.51/62.86	
D	T-Rha	5.17/109.75	4.51/81.89	4.15/83.68	4.03/79.89	3.62/70.8	1.19/19.45
E	1,3,4-GalpA	5.17/103.1	4.01/79.41	4.40/80.55	4.97/75.85	5.11/74.45	173.65
F	1,3,6-Gal*p*	5.33/103.27	4.03/73.41	4.40/79.41	4.11/77.92	4.40/75.84	3.76/62.86
G	T-GalpA	5.12/109.75	4.02/79.89	4.15/77.60	4.05/74.35	5.11/73.01	173.76
H	T-Galp	5.27/103.1	4.03/73.41	4.47/75.84	4.15/70.18	3.76/71.54	3.50/63.99
L	1,5-Ara*f*	5.11/110.39	4.13/81.89	3.82/80.55	3.67/70.80	3.51/63.99	

## Data Availability

The original contributions presented in this study are included in the article/Supplementary Materials. Further inquiries can be directed to the corresponding authors.

## Supplementary Materials

The following supporting information can be downloaded at: https://www.mdpi.com/article/10.3390/foods15071152/s1, Table S1 MTT assay results for BRP-1 treatment in HepG-2 cells; Table S2 MTT assay results for BRP-2 treatment in HepG-2 cells; Table S3 MTT assay results for BRP-3-1 treatment in HepG-2 cells; Table S4 Determination of glucose metabolism indicators in HepG-2 cells; Table S5 Determination of lipid metabolism indicators in HepG-2 cells; Table S6 Glucose standard curve preparation; Table S7 Protein standard curve preparation; Table S8 Preparation of standard curve of glyuronic acid.
