# Supplementary material for: Isolation, Purification, Structural Characterization of Acidic Polysaccharides from Brassica rapa L. Rhizomes and Their In Vitro Activity Verification in Ameliorating Glycolipid Metabolism Disorders
Source: Foods. 2026 Mar 27;15(7):1152. doi: 10.3390/foods15071152 (PMC13073851; doi:10.3390/foods15071152)
Supplement: Supplementary file 1 [file foods-15-01152-s001.zip › foods-4176525-supplementary.pdf]

Table S1 MTT assay results for BRP-1 treatment in HepG-2 cells（n=3）

Concentration（μg/mL）	cell viability 1（%）	cell viability 2（%）	cell viability 3（%）	Average（%）
800	96.90	109.25	98.31	101.49
400	89.40	90.43	95.00	91.61
200	86.30	83.53	101.46	90.43
100	87.17	86.73	103.69	92.53
50	91.47	86.46	97.60	91.84
25	90.80	84.10	95.97	90.29

Table S2 MTT assay results for BRP-2 treatment in HepG-2 cells（n=3）

Concentration（μg/mL）	Cell viability 1（%）	Cell viability 2（%）	Cell viability 3（%）	Average（%）
800	83.30	86.31	88.81	86.14
400	84.91	85.82	85.49	85.41
200	89.66	89.34	81.35	86.79
100	90.62	91.43	79.29	87.12
50	91.44	89.81	89.16	90.14
25	99.99	101.31	104.29	101.86

Table S3 MTT assay results for BRP-3-1 treatment in HepG-2 cells（n=3）

Concentration（μg/mL）	Cell viability 1（%）	Cell viability 2（%）	Cell viability 3（%）	Average（%）
800	101.88	106.18	112.58	106.88
400	103.21	101.61	111.01	105.28
200	91.52	87.67	106.76	95.32
100	89.73	88.36	108.56	95.55
50	91.01	90.30	96.15	92.49
25	93.31	97.52	101.87	97.57

Table S4 Determination of glucose metabolism indicators in HepG-2 cells (n=4)

Groups	Glucose consumption /mmol/L	Groups	Glucose consumption /mmol/L
Control	6.41	BRP-1_M	5.57

Control	6.80	BRP-1_M	6.99
Control	6.59	BRP-2_M	7.48
Control	6.47	BRP-2_M	6.3
Model	4.16	BRP-2_M	5.77
Model	3.12	BRP-2_M	5.54
Model	2.01	BRP-3-1_M	8.28
Model	1.64	BRP-3-1_M	7.65
BRP-1_H	6.13	BRP-3-1_M	7.18
BRP-1_H	5.45	BRP-3-1_M	7.08
BRP-1_H	4.85	BRP-1_L	6.19
BRP-1_H	4.47	BRP-1_L	5.83
BRP-2_H	6.50	BRP-1_L	5.26
BRP-2_H	5.05	BRP-1_L	5.11
BRP-2_H	5.51	BRP-2_L	6.67
BRP-2_H	4.10	BRP-2_L	5.73
BRP-3-1_H	8.57	BRP-2_L	5.18
BRP-3-1_H	7.80	BRP-2_L	5.02
BRP-3-1_H	7.56	BRP-3-1_L	7.50
BRP-3-1_H	7.85	BRP-3-1_L	6.21
BRP-1_M	5.37	BRP-3-1_L	7.43
BRP-1_M	5.05	BRP-3-1_L	8.38

Table S5. Determination of lipid metabolism indicators in HepG-2 cells (n=4)

Groups	TC /mmol/L	TG /mmol/L	Groups	TC /mmol/L	TG /mmol/L
Control	150.94	60.13	BRP-1_M	275.07	212.91
Control	152.87	67.85	BRP-1_M	236.03	132.41
Control	126.56	66.72	BRP-2_M	171.19	181.16
Control	152.19	68.99	BRP-2_M	153.56	84.22

Model	240.35	115.65	BRP-2_M	166.13	110.78
Model	232.17	129.16	BRP-2_M	174.34	128.45
Model	234.65	130.17	BRP-3-1_M	166.13	86.72
Model	217.67	134.61	BRP-3-1_M	161.11	79.81
BRP-1_H	202.00	87.98	BRP-3-1_M	161.11	70.16
BRP-1_H	207.02	165.79	BRP-3-1_M	166.13	87.17
BRP-1_H	167.41	90.02	BRP-1_L	229.77	113.7
BRP-1_H	223.94	93.09	BRP-1_L	214.59	95.12
BRP-2_H	159.22	79.68	BRP-1_L	233.5	81.72
BRP-2_H	175.59	84.79	BRP-1_L	232.23	88.07
BRP-2_H	174.95	90.92	BRP-2_L	213.34	107.35
BRP-2_H	166.77	82.64	BRP-2_L	229.06	105.1
BRP-3-1_H	156.08	73.08	BRP-2_L	232.23	99.07
BRP-3-1_H	162.39	77.18	BRP-2_L	236.62	104.2
BRP-3-1_H	157.98	73.66	BRP-3-1_L	190.15	117.45
BRP-3-1_H	161.76	73.44	BRP-3-1_L	213.38	99.62
BRP-1_M	209.69	91.37	BRP-3-1_L	210.22	106.78
BRP-1_M	230.37	108.26	BRP-3-1_L	207.08	97.29

Table S6. Glucose standard curve preparation

Glucose concentration （mg/mL）	0.0	0.12	0.24	0.36	0.48	0.60
**Standard glucose solution (μL)**	0	10	20	30	40	50
**Deionized water (μL)**	50	40	30	20	10	0
**Anthrone-sulfuric acid solution (μL)**	200					
React in a 96-well plate for 5 minutes, and measure the absorbance at 620 nm (n=3).						

Table S7 Protein standard curve preparation

BSA concentration (mg/mL)	0	0.02	0.04	0.06	0.08	0.10
**BSA solution (μL)**	0	10	20	30	40	50
**Deionized water (μL)**	50	40	30	20	10	0
**Coomassie Brilliant Blue (μL)**	250					

React in a 96-well plate for 5 minutes, and measure the absorbance at 595 nm (n=3)

Table S8 Preparation of standard curve of glyuronic acid

**D-Gal concentration（μg/mL）**	0	10	20	30	40	50	60	70	80	90
**D-Gal solution（μL）**	0	10	20	30	40	50	60	70	80	90
**Deionized water (μL)**	100	90	80	70	60	50	40	30	20	10
**Sodium tetraborate solution (μL)**	600									
Shake well, heat in a boiling water bath for 12 minutes, and cool to room temperature.										
**m-Hydroxybiphenyl (μL)**	8									
Mix thoroughly, react at room temperature for 40 minutes, and measure the absorbance at 525 nm.										
